# Virtual reality in chemotherapy support for the treatment of physical functions, fear, and quality of life in pediatric cancer patients: A systematic review and meta-analysis

**DOI:** 10.3389/fpubh.2023.1039720

**Published:** 2023-04-12

**Authors:** Oliver Czech, Sebastian Rutkowski, Aleksandra Kowaluk, Paweł Kiper, Iwona Malicka

**Affiliations:** ^1^Department of Physiotherapy, Wroclaw University of Health and Sport Sciences, Wroclaw, Poland; ^2^Department of Physical Education and Physiotherapy, Opole University of Technology, Opole, Poland; ^3^Department of Pediatric Bone Marrow Transplantation, Oncology, and Hematology, University Hospital, Wroclaw, Poland; ^4^Healthcare Innovation Technology Lab, IRCCS San Camillo Hospital, Venice, Italy

**Keywords:** virtual reality, childhood cancer, chemotherapy, pediatric patients, pain, anxiety, fear

## Abstract

**Background:**

Appropriately selected complementary therapies, such as virtual reality (VR) and active video games (AVG), provide support to young patients during the process of cancer treatment. Therefore, this systematic review with meta-analysis aimed to analyze the effects of VR and AVG on fear, physical functions, and quality of life.

**Methods:**

A systematic search was performed independently in Scopus, PubMed, Embase, Web of Science and Cochrane Library electronic databases for relevant randomized controlled and crossover studies. From a total of 5,963 records, 11 met the inclusion criteria. After full-text screening two publications were excluded, yet six studies were included in the quantitative analysis because three studies had a large discrepancy in their measured outcomes. For methodological quality assessments, the RoB2 software program was used, while RevMan 5.4.1 was used for statistical analysis and meta-analysis. Standard Mean Difference (SMD) outcome measures were used for the analysis. Statistical heterogeneity was assessed using the I2 statistic with a cut-off value of 50% considering intervention and outcome measures.

**Results:**

Our systematic review includes six randomized controlled studies and three randomized crossover studies. The participants represented both sexes and were children and adolescents (<18 years old) with a diagnosis of cancer. The analysis of the results allows for a careful conclusion that VR has the potential to become an accessory tool in rehabilitation and oncologic treatment. All of the included studies noted a significant advantage of this intervention.

**Conclusion:**

VR has the potential to be an effective and important tool in the oncologic treatment of children. VR immerses the patient, and as a result, produces a distraction that effectively reduces pain associated with standard oncologic care procedures in children. However, this systematic review and meta-analysis highlights the need for more research into the use of VR as support for pediatric oncologic care.

**Systematic review registration:**

PROSPERO database (https://www.crd.york.ac.uk/prospero/display_record.php?RecordID=319000), CRD42022319000.

## 1. Introduction

A steady increase in the incidence of some malignant neoplasms, mostly hematologic, has been observed in the general population and children and adolescents. The dynamic development of diagnostic and therapeutic approaches has contributed to an increase in the percentage of children cured and achieving a complete remission of the disease (1, 2). Therefore, the efficiency of treatments and activity of these patients, as well as their mental wellbeing and quality of life, both during treatment and in early adulthood, are of increasing importance. However, hospitalizations, invasive examinations, lack of contact with peers, and subordinating life to their illness are the main causes of mental malaise in children with cancer.

The most common adverse physical effects of antineoplastic therapy (e.g., chemotherapy) include general fatigue, cardiovascular disorders, decreased tolerance to exercise, weight fluctuations, osteopenia, myopathy, neuropathy, as well as damage to the central nervous system (3). These symptoms translate into a reduction in daily physical activity and may contribute to the development of anxiety disorders and stress, and therefore lead to deterioration of sleep quality (4).

Anxiety and stress in children are also generated by medical procedures. The treatment course is associated with several repetitive and unpleasant procedures, such as venous port access, venipuncture, tissue biopsy, and bone marrow puncture. Most of the treated children describe such procedures as one of the most disturbing and stressful aspects of the entire treatment process of their illness (5).

Stress experienced by children due to a cancer diagnosis affects the psychological wellbeing of these children. It has been proven that psychological stimuli can affect the sensation of pain, and as such, mental and physical suffering is associated with feelings of severe pain (6–11). Current studies suggests that stress, anxiety, and fear can increase pain as well as be its source (12, 13). The World Health Organization (WHO) published recommendations for the pharmacological treatment of a child's pain, acknowledging that pain in children is a public health concern of high significance in the whole world [(14). Available from: https://www.ncbi.nlm.nih.gov/books/NBK138354/]. Pain also significantly affects quality of life. Consequently, the development of a child's psychological and social sphere, which occurs in the period of early childhood, becomes disrupted (15, 16).

Appropriately selected complementary therapies, such as virtual reality (VR) and active video games (AVG), provide support to young patients during the process of cancer treatment (17). VR refers to the real-time simulation of an interactive environment, scenario, or activity. The varying degrees of user immersion in VR have led to its categorization into non-immersive, immersive, mixed, and augmented reality, each characterized by differences in the number of virtual stimuli presented and the extent to which real-world stimuli are suppressed (18, 19). AVGs, also known as “exergames,” are defined as video games incorporating movement or in which movement is encouraged by a game controller, which may include motion-responsive cameras or handheld versions, mats or boards (20). It is recommended to seek and develop novel cognitive-behavioral interventions utilizing VR and AVG to distract from these painful procedures. Methods to reduce the sensory and affective components of pain along with the ability to distract attention have been shown to be an effective strategy in minimizing procedural pain, anxiety, and stress. Moreover, distraction can also be a tool for modifying the way pain stimuli are processed (21).

In recent years, several studies have demonstrated a positive effect of combining psychological interventions with pharmacological treatments to reduce pain (22). These methods aim to reduce negative consequences of neoplastic diseases on the mental state and quality of life of children and adolescents (23). Therefore, this systematic review with meta-analysis aimed to investigate the effects of VR and AVG on fear, physical functions, and quality of life.

## 2. Methods

This review followed the Preferred Reporting Items for Systematic Reviews and Meta-Analyses 2020 (PRISMA) guidelines (24). Extraction of data was performed using the template for intervention description and replication (TIDieR) framework for the reporting of interventions (see Supplementary material 3). The protocol was prospectively registered in the PROSPERO database (CRD42022319000).

### 2.1. Electronic search

Two researchers independently analyzed the following databases for relevant research articles: PubMed, Web of Science, Scopus, Embase, and Cochrane. The search was performed in March and April of 2022. There was no time frame for publication set as the number of publications on this topic is limited. The search strategy was based on the following medical subject headings (MeSH Terms): “children,” “teenager,” “adolescents,” “pediatric,” “neoplasms,” “tumor,” “cancer,” “malignant,” “neoplasm,” “Leukemia,” “Lymphoma,” “Leucocythemia,” “virtual reality,” “VR,” “video game,” “exergaming,” “AVG,” “IVG,” “Xbox,” “interactive video game,” “wii,” “Kinect,” “physical activity,” “motor performance,” “fatigue,” “body coordination,” “energy expenditure,” “core executive functions,” “pain,” and “fear.” The search strategy description is presented in the Supplementary material (see Supplementary material 1).

### 2.2. Study selection

The inclusion criteria concerned randomized controlled trials including crossover studies. Articles in English were sought. Google Scholar databases and the reference lists of these articles were also analyzed for gray literature search items. Two authors independently screened each abstract using the inclusion/exclusion criteria template. All differing opinions were resolved by a third investigator. The quality analysis of full-text articles (risk of bias assessment) was then performed according to the same procedures. Eligibility criteria were defined using the PICO Framework (25).

- *P*—children and adolescents (<18), undergoing cancer treatment, regardless of cancer type;
- *I*—treatment with AVG and/or VR (immersive, non-immersive, or mixed-reality) to provide distraction during diagnostic and therapeutic procedures;
- *C*—standard rehabilitation;
- *O*—Primary: physical activity, motor performance, core executive functions, Secondary: fear, pain, fatigue, body coordination, energy expenditure.

### 2.3. Outcomes

This systematic review and meta-analysis aimed to analyze the effect of VR and AVG on physical functions, fear, and quality of life. Outcomes such as fear, pain, fatigue, body coordination, EE, physical activity, motor performance, and core executive functions were analyzed in a controlled and experimental environment. The experimental AVG and/or VR treatment group was compared to standard-of-care or no-intervention control groups.

### 2.4. Data extraction and management

Data extraction was carried out by two reviewers. Relevant data, such as authors, publication date, study design, participants' characteristics, co-interventions, sample size, intervention type, outcome measurement, and dates of administration were provided on a data extraction form.

### 2.5. Assessment of risk of bias in the selected studies

For methodological quality assessments, the RoB2 software program (Risk of Bias, version 2.0 Cochrane Collaboration, 2020) was used. The quality of the studies was judged based on the bias categories of selection bias (sequence generation and allocation concealment), detection bias (blinding of outcome assessment), attrition bias (incomplete outcome data), reporting bias (selective reporting), and all other biases. An additional category was used to describe crossover studies such as bias arising from period and carryover effects (labeled D1b in **Figure 3**). The risk of bias was assessed on a three-point scale, where “low” stands for a low possibility of bias, “high” means a high possibility of bias, and “some concerns” is used when the manuscript does not contain valuable information. In cases where risk was not described in the text, the authors were contacted two times (2 weeks apart) to complete the missing data.

### 2.6. Data synthesis and statistical analysis

For statistical analysis and meta-analysis calculations RevMan 5.4.1 was used. It was attempted to categorize the interventions into three outcome groups: (1) pain, (2) fear, and (3) anxiety. Standard Mean Difference (SMD) outcome measures were used for the analysis since the selected studies used different tools. Statistical heterogeneity was assessed using the *I*^2^ statistic with a cut-off value of 50% considering intervention and outcome measures. Our meta-analysis was based on a random model with a 95% confidence interval. In the event that no data were available for analysis, the corresponding authors were contacted two times. We waited for 2-weeks to receive a response. Due to the limited number of studies, it was not possible to perform subgroup analysis in cases of high heterogeneity.

## 3. Results

**Figure 1:**
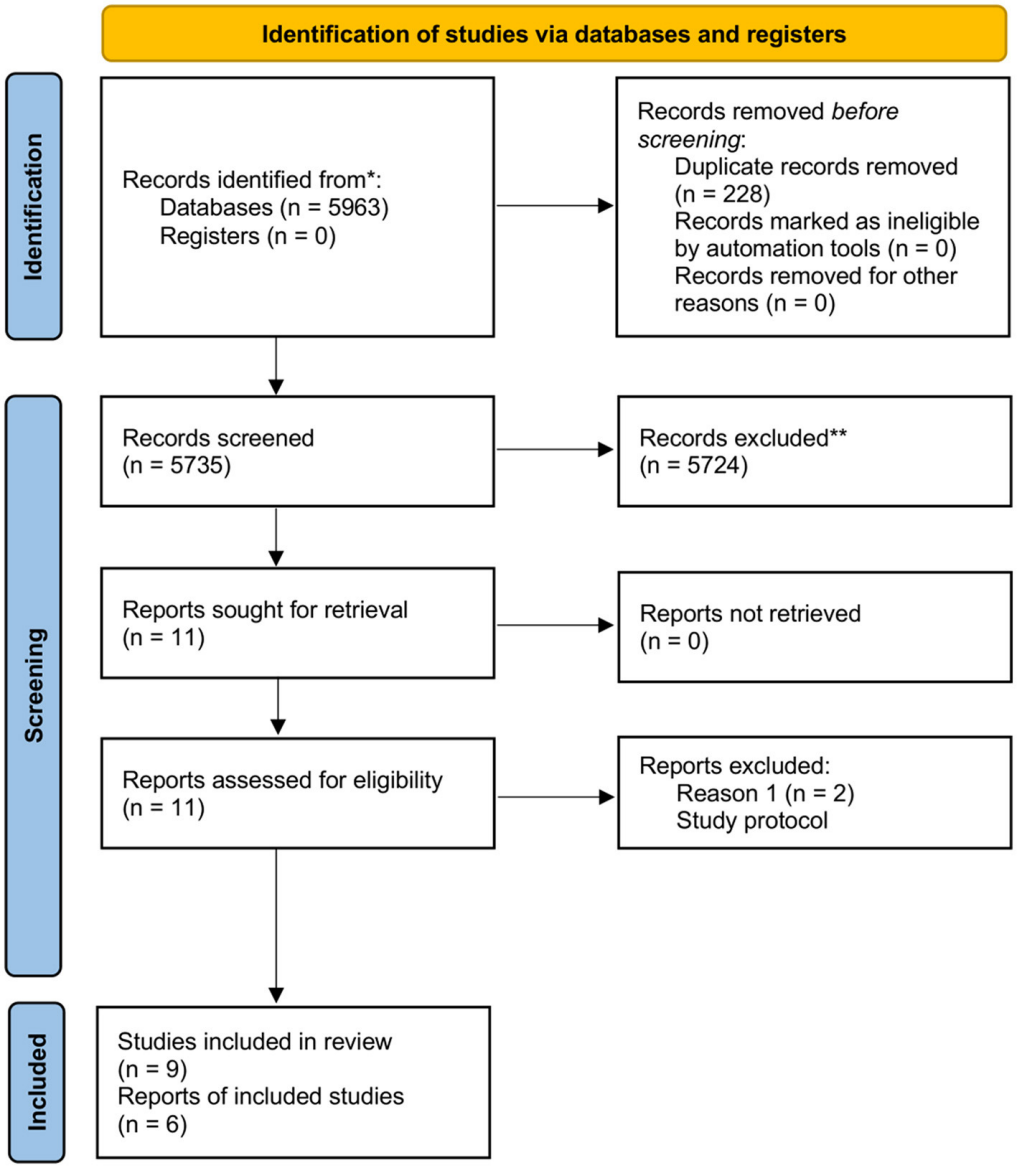
PRISMA flowchart for review process.

The electronic search identified 5,963 overall search items with no additional records from our gray literature analysis. The following numbers of publications were sought from each of the databases: PubMed: 76, Web of Science: 43, Scopus: 5,665, Embase: 126, and Cochrane: 53. The 228 duplicate records were deleted, leaving a total of 5,735 abstracts for screening. At this stage, 5,724 records did not meet the inclusion criteria and therefore were excluded. The main reasons for removing the abstracts were: other as agreed study design, no VR intervention during chemotherapy, different study populations, and publication type other than articles. Finally, 11 full-text articles were evaluated for eligibility, of which 9 studies met the inclusion criteria and classified for qualitative analysis. The reason for exclusion of the other two full-text articles was a lack of outcomes as they were only study protocols. Six studies were included in the quantitative analysis because three studies had a large discrepancy in their measured outcomes. The review process is presented in a PRISMA flowchart (Figure 1).

### 3.1. Studies included in the review

Our systematic review included six randomized controlled studies and three randomized crossover studies. The participants represented both sexes and were children and adolescents (<18 years old) with a diagnosis of cancer. The age of the patients was between 6–18 years old. The term chemotherapy was assumed without a distinction regarding the type, and studies using supplemental radiotherapy were also included. The different types of childhood cancer treated in the included studies were neoplasms, tumors, leukemia, lymphoma, and leucocythemia.

Benzing et al. assessed the effects of cognitive exercise and exergaming in pediatric cancer patients. The researchers evaluated the effects of the intervention using three comparison groups (working memory exercise, exergaming, and a wait-list control condition) on cognitive efficiency in pediatric cancer patients. This study included 69 cancer patients. The children were randomly assigned to an 8-week work memory training, exergaming, or a wait-list control group. Each participant had to perform 345-min training sessions per week. The primary outcome evaluated was core executive functions. The secondary outcomes included other cognitive domains, motor functionality, and a parent rating on their children's executive functions. Measures were conducted before and after interventions and in a follow-up study 3 months later. According to the authors, there can be several conclusions drawn: working memory training improves visual working memory in pediatric cancer survivors. Also, near-transfer, but no far-transfer effects can be expected from working memory training. Additionally, multiple-component interventions tailored to fit the individual's cognitive profile are needed to best support cognitive development after cancer and its treatment (26).

Gerçeker et al. investigated the effect of VR distraction while accessing venous ports with a Huber needle. The study assessed needle-related pain levels, as well as fear, and anxiety in children and adolescents with cancer. This study included 42 patients. The researchers designed a parallel trial following the CONSORT checklist. The primary outcomes were patient-reported pain scores after the procedure, as well as fear and anxiety scores before and after the intervention. According to the authors, VR is an effective distraction method in reducing port needle-related pain, fear, and anxiety in pediatric patients (27).

The study by Gold and colleagues attempted to verify if VR treatment reduces pain and anxiety outcomes in patients during peripheral intravenous catheter (PIVC) placement. The effects of VR were compared to a standard care group. The study was carried out in 2 clinical wards: a radiology unit and an infusion center. This study included 107 patients. Children were randomized into standard care (simple distraction techniques) or VR with standard care groups. Primary outcomes included pain and anxiety reported by the patient, caregiver, and clinician during PIVC placement. The results were investigated using generalized linear modeling with step-backward selection for the final model construction. Authors assessed VR pain distraction for IV Placement as feasible and useful in an outpatient radiology department (28).

A publication by Hundert et al. assessed the effects of VR distraction on decreasing procedural pain during subcutaneous port (SCP) pediatric cancer patients. This study included 40 patients. Due to logistic challenges outlined below, the trial was subsequently modified to a parallel 2-arm design with participants being randomly assigned to the study group for 1 SCP needle insertion only. The result showed, VR as a distraction intervention was feasible and acceptable to patients, as well as their families, and clinicians (29).

Sabel et al. (30) evaluated whether AVG improves body coordination in survivors of childhood brain tumors. This study included 13 patients. The children were randomly assigned to AVG or waiting list groups with 10–12 weeks of crossover. Children during AVG training played for min. 30 min per day, 5 days a week for 10 weeks, but were allowed to extend the period to 12 weeks to compensate for weeks being away or ill. Weekly online coaching sessions were held to maintain motivation and evaluate enjoyment. The researchers assessed EE levels and physical functioning in single-blinded assessments using the Bruininks-Osteretsky test of motor performance. The secondary outcomes evaluated participants before and after the intervention, as well as, compared the randomization groups after the first period. In this group of childhood brain tumor survivors, home-based AVG, supported by a coach, was a feasible, enjoyable and moderately intense form of exercise that improved Body Coordination (30).

In Gold et al. (28) evaluated the effects of physical AVG on cognition and activities of daily living. They conducted a randomized controlled pilot study in a group of childhood brain tumor survivors. This study included 23 patients. The children were randomly assigned to the intervention and waiting list groups. After 10–12 weeks the groups were changed using crossing over design. The intervention consisted of AVG using a motion-controlled video console (Nintendo Wii) for 30 min a day, at least 5 days a week, and weekly web-based coaching sessions. Meetings and measurements before and after each period included tests regarding the performance of activities of daily living using the Assessment of Motor and Process Skills (AMPS) and cognitive tests. The test pre- and post-intervention scores were compared. Following conclusions are drown by the authors: active video gaming used as a home-based intervention for childhood brain tumor survivors improved motor and process skills in activities of daily living (31).

Semerci et al. evaluated the effect of VR on pain during venous port access in pediatric cancer patients. This study included 71 patients. The children were randomly assigned to VR intervention and control groups. Only standard care was used in the control conditions. A VR headset was fitted to the children in the experimental group. After port access, pain scores were obtained from the child's self-report and the parents' proxy report using the Wong-Baker FACES Pain Rating Scale. According to the results, the use of VR for children receiving blood draw is an effective non-pharmacological method to decrease pain, fear and anxiety (32).

A study by Sharifpour et al. assessed the impact of VR therapy on perceived pain intensity, anxiety, catastrophizing, and self-efficacy in adolescents with cancer. This study included 30 patients. Patients were randomly assigned to experimental and control settings. The intervention group underwent 830-min sessions of VR therapy once a week for 2 months. The control group was put on a waiting list. The primary outcome included pain measurements. The authors are stating, VR can improve pain-related variables among adolescents with cancer during chemotherapy (33).

Wong et al. evaluated whether VR intervention reduced pain and anxiety in children with cancer. This study included 108 patients. The experimental group received VR distraction and as a control environment, standard care procedures were used. The primary outcome was pain as reported by the children. Secondary outcomes were anxiety, pulse rate, and procedure duration. Outcome measurements were conducted before, during, and immediately after the procedure. The study leads to a conclusion, using VR in clinical setting is feasible and effective in reducing pain and anxiety among pediatric patients undergoing PIC (34).

A detailed description of the characteristics of included studies is included in Supplementary material 2.

### 3.2. Excluded studies

Two studies were excluded after the full-text analysis. The results shown by Benzing et al. (35) and Kauhanen et al. (36) were insufficient compared to the rest of the data due to a lack of results (study protocols).

### 3.3. Risk of bias in the studies

**Figure 2:**
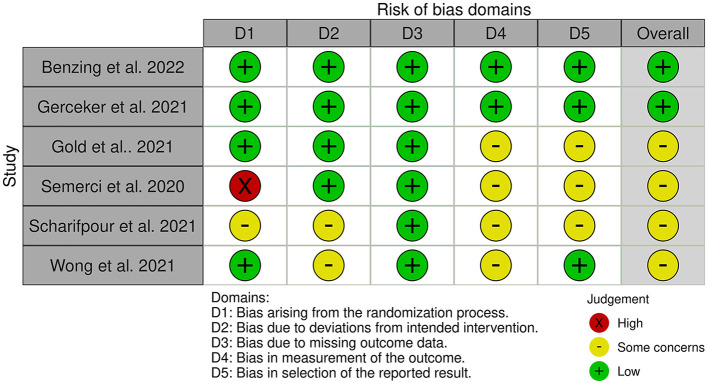
Risk of bias assessment of RCT studies.

**Figure 3:**
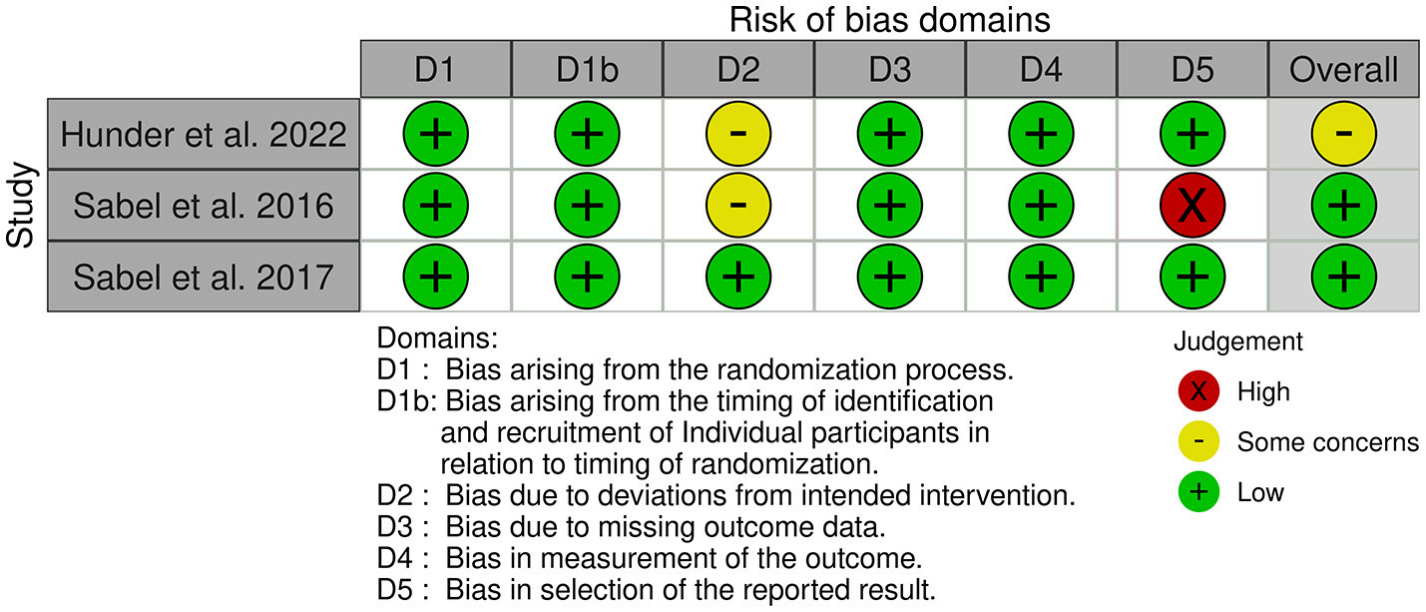
Risk of bias assessment of crossover studies.

Figures 2, 3 show the risk of bias for the publications included in the review. The articles were judged using 2 different templates (tool to implement ROB 2 and tool to implement ROB 2 for crossover studies) due to the use of crossover studies (Figures 2, 3).

- Randomization process: Four RCTs studies (26–28, 34) and all crossover (29– 31) was assessed as a low risk of bias. The authors described in detail the randomization component of the sequence-generation process. One study (33) had an unclear risk of bias due to no information about the randomization was provided. One Semerci et al. (32) of the studies did not have randomized control conditions and was assessed as having a high risk of selection bias.
- Bias arising from period and carryover effects: All crossover studies (29–31) had a low risk of bias in this domain.
- Deviations from intended interventions: Four RCTs (26–28, 32) and one crossover (31) study had a low risk of bias. As there was no information about deviations, two RCTs (33, 34) and two crossover (29, 30) studies were assessed as having an unclear risk of bias.
- Missing outcome data: All studies (26–34) had complete data sets.
- Measurement of the outcome: Two RCTs (26, 27) and all crossover studies (29–31) were evaluated as having a low risk of bias. Four RCTs (28, 32–34) were judged to have an unclear risk of bias due to missing information.
- Selection of reported results: Three RCTs (26, 27, 34) and two crossover studies (29, 31) were assessed as having a low risk of bias. Three RCTs (28, 32, 33) were assessed as having an unclear risk of bias. One crossover study (30) was assessed as having a high risk of bias.
- The reported results of the analysis show that three randomized controlled trials (26, 27, 34) and two crossover studies (29, 31) had a low risk of bias. Meanwhile, three randomized controlled trials (28, 32, 33) were deemed to have an unclear risk of bias. Finally, one crossover study (30) was assessed as having a high risk of bias.

### 3.4. Effects of intervention

#### 3.4.1. Comparison of VR treatment and standard oncologic treatment: Pain outcome

**Figure 4:**
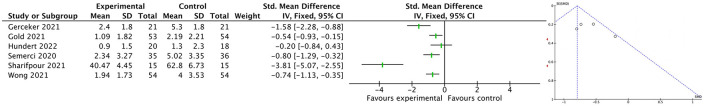
Funnel plot of comparison 1.

With a total of 324 participants, six studies were analyzed for pain scores during standard oncologic care procedures. In all of the studies, immersive VR was used as an experimental intervention. Due to the different pain scales used in the included studies, the analysis was performed using SMD with a random model effect. The meta-analysis showed a significant difference between the two treatment conditions in comparison (SMD = −1.08; 95% CI [−1.65 to −0.51]; *I*^2^ = 84%). Despite statistical significance, these studies were too heterogeneous to be pooled. Figure 4 shows the funnel plot of comparison 1.

#### 3.4.2. Comparison of VR treatment and standard oncologic treatment: Anxiety outcome

**Figure 5:**
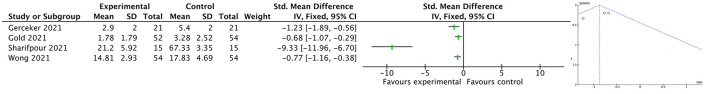
Funnel plot of comparison 2.

With an overall number of 286 participants, four studies were analyzed for anxiety levels during standard oncologic care procedures. In all of the studies, immersive VR was used as the experimental intervention. The analysis was performed using SMD with a random model effect. The meta-analysis showed a significant difference between the two treatment conditions in comparison (SMD = −1.86; 95% CI [−2.98 to −0.73]; *I*^2^ = 93%). Despite statistical significance, these studies were too heterogeneous to be pooled. Figure 5 shows the funnel plot of comparison 2.

#### 3.4.3. Comparison of VR treatment and standard oncological treatment: Fear outcome

**Figure 6:**
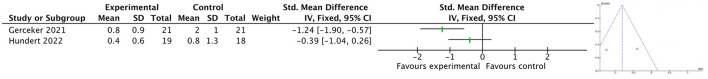
Funnel plot of comparison 3.

With an overall number of 79 subjects, two studies were analyzed for fear levels during standard oncologic care procedures. In both studies, immersive VR was used as the experimental intervention. In this comparison, the analysis was performed using SMD with a random model effect. The meta-analysis showed no significant difference between the two treatment conditions in comparison (SMD = −0.81; 95% CI [−1.64 to −0.02]; *I*^2^ = 69%). However, the results were too heterogeneous to be pooled. Figure 6 shows the funnel plot of comparison 3.

## 4. Discussion

This systematic review with meta-analysis aimed to analyze the effectiveness of VR and AVG on physical functions, pain, fear, and anxiety. The analysis of the results allows for a careful conclusion that VR has the potential to become an accessory tool in rehabilitation and oncologic treatment. However, due to the large methodological differences in the analyzed publications, conclusions should be drawn with great caution. Although the benefits for motor function resulting from the use of VR in oncologic treatment were not investigated, there were clear trends toward improved mental health. The improvement in pain and anxiety parameters were noticeable not only in the overall results but also in almost every study separately. However, due to insufficient data, physical function outcomes could not be analyzed. These results should encourage researchers to undertake research in this direction and justify why research on VR should be continued. Subsequent research with the use of VR should focus largely on the standardization of the research methodology, which will help to improve the effectiveness in evaluating this tool.

Current scientific research using VR in oncology focuses primarily on parameters of mental health. This is mainly due to the potential of this technology. It is noticeable that research into the impact of VR and AVG technologies on physical parameters is gaining popularity. Analyzing the published research topics in the last years it seems probable that in the future, scientists will discover a lack of studies evaluating a new application of modern technology. However, analysis of the included works allowed us to assess the effectiveness of VR and AVG as complementary therapies that supports analgesic and anxiolytic activities and reduces procedural fear.

The review of the articles identified two groups of studies in terms of the used intervention. In three articles (26, 30, 31), the experimental intervention AVG was used, while in the other articles the researchers used VR. Unfortunately, the AVG studies could not be included in the meta-analysis according to a large discrepancy in the measured results. The quality of the analyzed studies was relatively high with occasional concerns. The potential of this modern technology could also be used in other fields of medicine. Although the results show statistically significant benefits of VR intervention, one should not draw overstated conclusions due to the high heterogeneity of the compared studies. The review supports the need for additional research in this field. Researchers should focus on analyzing larger patient groups and standardizing the methodology.

The trends noticed are in line with the results of a mini-review (37), narrative review (38), and meta-analysis (39). VR is an effective tool in the pain management and distraction of cancer patients, and may also reduce additional emotional side effects. A meta-analysis by Zeng et al. showed a positive effect of VR-based interventions on reducing patients' anxiety (SMD = −3.03; 95% CI [−6.20 to 0.15]). Also, our results noted a very high heterogeneity (*I*^2^ = 95%) rendering the results unreliable. The overall outcomes favored a VR intervention for fatigue and pain level reduction, but only fatigue symptoms were statistically significant (MD = −2.50; 95% CI [−5.97 to −0.99]; *I*^2^ = 16%).

Mental health and mental wellbeing are important factors in cancer rehabilitation, as they directly affect the general rehabilitation process. The diagnosis, treatment, hospitalization, and prognosis of oncologic diseases generate stress, fear, and anxiety leading to a decrease in overall physical activity (40). Mental symptoms affect patients during all stages of the disease. Stress has also been shown to cause patients to tolerate therapy less and to be less cooperative with medical personnel (41). Most studies agree that distraction is an attempt to justify the mechanism for the effectiveness of modern technologies. Although, for some researchers, reducing pain seems to be a placebo effect. According to Buhle et al. (42), placebo and distraction provide two pathways for pain relief. The authors have monitored and analyzed functional MRI imaging. The results suggest distraction effectively inhibits pain processing in the central nervous system, while a placebo may not influence pain processing. It seems that the pain-relief effect during chemotherapy results in reduced anxiety. Such a hypothesis was confirmed by our team in a previous meta-analysis on VR interventions and needle-related procedural pain, fear, and anxiety (43), likewise in the study on burn wound care (44). Statistically significant benefits of VR interventions were shown in children's pain levels, where VR significantly decreased symptoms (*n* = 3,204 patients, MD = −2.85; 95% CI [−3.57 to −2.14]) as measured by the Wong-Baker Faces Pain Rating Scale and by the Faces Pain Scale-Revised (*n* = 2,240 patients, MD = −0.19; 95% CI −0.58 to 0.20).

## 5. Conclusion

VR has the potential to be an important tool in the oncologic treatment of children. VR, by using the immersion phenomenon produces a distraction that effectively reduces pain associated with standard oncologic care procedures in children. All of the investigated studies noted a significant advantage of this type of intervention. Thus, VR could increase psychophysical wellbeing, especially in pediatric patients. However, this systematic review and meta-analysis highlights the need for more studies into the use of VR as support for pediatric oncologic care. Research on larger groups, with similar conditions, could provide evidence of the effectiveness of VR distraction interventions and enable the inclusion of such intervention into standard medical procedures.

While VR shows promise as a valuable tool in the oncologic treatment of children, it is important to exercise caution when drawing conclusions. Through immersion, VR can effectively reduce pain associated with standard oncologic care procedures in children, as noted by all investigated studies. This intervention has the potential to improve the psychophysical wellbeing of pediatric patients. However, this systematic review and meta-analysis highlight the need for further research into the use of VR as a support for pediatric oncologic care. Conducting studies with larger sample sizes and similar conditions could provide evidence of the effectiveness of VR distraction interventions and facilitate their integration into standard medical procedures.

## Data availability statement

The raw data supporting the conclusions of this article will be made available by the authors, without undue reservation.

## Author contributions

OC and IM contributed to the design and implementation of the study. OC, SR, and AK contributed to analysis, interpretation of data, and were involved in drafting the manuscript and contributed to the assessing quality of studies. PK and IM contributed to the interpretation of data and were involved in revising the manuscript. OC and AK contributed to the data extractions and data management. All authors read and approved the final manuscript.
