# Supplementary material for: Virtual reality in chemotherapy support for the treatment of physical functions, fear, and quality of life in pediatric cancer patients: A systematic review and meta-analysis
Source: Front Public Health. 2023 Apr 12;11:1039720. doi: 10.3389/fpubh.2023.1039720 (PMC10130570; doi:10.3389/fpubh.2023.1039720)
Supplement: Supplementary file 1 [file Data_Sheet_1.docx]

Supplementary_Material_1. Search strategy

**PUBMED**

**#1** (children OR teenager OR adolescents OR pediatric)

**#2** ("neoplasms"[MeSH] OR Neoplas* OR Tumor* OR Cancer* OR Malignan* OR "Malignant Neoplasm*" OR "Neoplasm, Malignant" OR Leukemia [MeSH] OR Lymphoma [MeSH] OR Leucocythaemia [MeSH])

**#3** ("virtual reality" OR VR OR "video game*" OR exergaming OR AVG OR IVG OR Xbox OR "interactive video game*" OR wii OR kinect)

**#4** ("physical activity" OR "motor performance" OR fatigue OR "body coordination" OR "energy expenditure" OR "core executive functions" OR pain OR fear)

**#5** (#1 AND #2 AND #3 AND #4)

TOTAL=76

**Web of Science**

**#1** WC= Oncology

**#2** WC= Pediatrics

**#3** WC= Rehabilitation

**#4** (#1 OR #2 OR #3)

**#5** TS=(children OR teenager OR adolescents OR pediatric)

**#6** TS=(neoplasms OR Neoplas* OR Tumor* OR Cancer* OR Malignan* OR "Malignant Neoplasm*" OR "Neoplasm, Malignant" OR Leukemia OR Lymphoma OR Leucocythaemia)

**#7** TS=("virtual reality" OR VR OR "video game*" OR exergaming OR AVG OR IVG OR Xbox OR "interactive video game*" OR wii OR kinect)

**#8** TS=("physical activity" OR "motor performance" OR fatigue OR "body coordination" OR "energy expenditure" OR "core executive functions" OR pain OR fear)

**#9** (#4 AND #5 AND #6 AND #7 AND #8)

TOTAL= 43

**Scopus**

**#1** children OR teenager OR adolescents OR pediatric

**#2** neoplasms OR Neoplas* OR Tumor* OR Cancer* OR Malignan* OR "Malignant Neoplasm*" OR "Neoplasm, Malignant" OR Leukemia OR Lymphoma OR Leucocythaemia

**#3** "virtual reality" OR VR OR "video game*" OR exergaming OR AVG OR IVG OR Xbox OR "interactive video game*" OR wii OR Kinect

**#4** “physical activity" OR "motor performance" OR fatigue OR "body coordination" OR "energy expenditure" OR "core executive functions" OR pain OR fear

**#5** (#1 AND #2 AND #3 AND #4)

TOTAL=5665

**Embase**

**#1** children OR teenager OR adolescents OR pediatric

**#2** neoplasms OR Neoplas* OR Tumor* OR Cancer* OR Malignan* OR "Malignant Neoplasm*" OR "Neoplasm, Malignant" OR Leukemia OR Lymphoma OR Leucocythaemia

**#3** "virtual reality" OR VR OR "video game*" OR exergaming OR AVG OR IVG OR Xbox OR "interactive video game*" OR wii OR Kinect

**#4** “physical activity" OR "motor performance" OR fatigue OR "body coordination" OR "energy expenditure" OR "core executive functions" OR pain OR fear

**#5** (#1 AND #2 AND #3 AND #4)

TOTAL=126

**Cochrane**

**#1** MeSH descriptor [neoplasms] explode all trees

**#2** MeSH descriptor [Leukemia] explode all trees

**#3** MeSH descriptor [Lymphoma] explode all trees

**#4** MeSH descriptor [Leucocythaemia] explode all trees

**#5** (Neoplas* OR Tumor* OR Cancer* OR Malignan* OR "Malignant Neoplasm*"):ti,ab,kw

**#6** (children OR teenager OR adolescents OR pediatric):ti,ab,kw

**#7** (“virtual reality” OR VR OR “video game* OR exergaming OR AVG OR IVG OR xbox OR “interactive video game” OR wii OR kinect):ti,ab,kw

**#8** (“physical activity” OR “motor performance” OR fatigue OR “body coordination” OR “energy expenditure” OR “core executive functions” OR pain OR fear):ti,ab,kw

**#9** #1 OR #2 OR #3 OR #4 OR #5

**#10** #9 AND #6 AND #7 AND #8

TOTAL=53
