# Supplementary material for: Virtual reality in chemotherapy support for the treatment of physical functions, fear, and quality of life in pediatric cancer patients: A systematic review and meta-analysis
Source: Front Public Health. 2023 Apr 12;11:1039720. doi: 10.3389/fpubh.2023.1039720 (PMC10130570; doi:10.3389/fpubh.2023.1039720)
Supplement: Supplementary file 2 [file Data_Sheet_2.docx]

Supplementary_Material_2. Characteristics of included studies

| Benzing et al., 2020 | |
| --- | --- |
| **STUDY DESIGN** | RCT |
| **PARTICIPANT** | Cancer survivors aged 7-16, >12 months after cancer treatment |
| **INCLUSION CRITERIA** | -age 7-16  -cancer diagnosis  -cancer treatment terminated at least 12 months before participation  -direct damage to the central nervous system or indirect consequences due to radio- or chemotherapy |
| **EXCLUSION CRITERIA** | -unstable health status  -unstable physical conditions  -patients unable to follow the study procedures (e.g., language problems) |
| **INTERVENTION ENVIRONMENT** | PC/Console, non-immersive VR |
| **INTERVENTION** | 1. Control group (n=24)  - Wait-list - No additional intervention  1. Exergaming (n=22)  - Kinect based - Exergaming application - 6 different workouts improving physical and cognitive demands  1. Working memory training (n=23)  - 13 tasks for visual and verbal working memory improvement - Home based, parental supervision - Trained psychologist monitoring   All of the interventions duration was 45 minutes training, 3 times a week, for 8 weeks. |
| **OUTCOMES** | 1. Primary outcome:  - 3 core executive functions - Block Recall Test of the Working Memory Test Battery for Children and the Color-Word Interference Test of the Delis-Kaplan Executive Function System  1. Secondary outcomes:   German version of the Kaufman Assessment Battery for Children—Second Edition:   - Fluid intelligence - Test of Nonverbal Intelligence - Verbal working memory – Number Recall, Word Order, - Planning – Rover - Verbal memory performance - Atlantis, Atlantis Recall   German Version of the Wechsler Intelligence Scale for Children, Fourth Edition:   - Selective attention – cancellation - Processing speed – Coding, Symbol Search   Executive functions in real-world context - Behavior Rating Inventory of Executive Functions  Motor ability - German Motor Test |
| **CONCLUSION** | -Working memory training improves visual working memory in pediatric cancer survivors.  -near-transfer, but no far-transfer effects can be expected from working memory training  -Multiple-component interventions tailored to fit the individual’s cognitive profile are needed to best support cognitive development after cancer and its treatment. |

| Gerçeker et al. 2021 | |
| --- | --- |
| **STUDY DESIGN** | Prospective RCT |
| **PARTICIPANT** | Patients from aged 6-17 undergoing Huber needle insertion into a subcutaneously implanted intravenous port. |
| **INCLUSION CRITERIA** | - patients from the hospital's Department of Radiology aged 6-17  -  undergoing Huber needle insertion  -  previous experiences of the Huber needle insertion. |
| **EXCLUSION CRITERIA** | -  aged <6 and >17 years  -  septic, dehydrated, vomiting, sedated, medically unstable,  -  previously known severe needle insertion phobia,  -  no Huber needle had ever inserted before  -  reported pain for another reason in the time of access the venous port with Huber needle  -  had cognitive impairment, or visual problem  -  children and parents that do not understand and speak Turkish |
| **INTERVENTION ENVIRONMENT** | - immersive VR |
| **INTERVENTION** | 1. Experimental group- standard care + VR (n=21)  -  Samsung Gear Oculus headset  -  Three VR applications were used in this study; swimming with marine animals underwater (Ocean Rift), riding a rollercoaster (Rilix VR), and exploring the forest through the eyes of woodland species (In the eyes of animal).  - the intervention began 2/3 minutes before the IV placement and ended when procedure has been completed  2. Control group- standard care (n=21)  -  patients and their parents were informed by the staff at least 1 h before the procedure,  - none pharmacological methods were used at the accessing the venous port with Huber needle |
| **OUTCOMES** | 1. Primary outcomes:  -  patient-reported pain scores  Measured using the  Wong-Baker FACES (WBS) Pain Rating Scale.  2. Secondary outcomes  - anxiety score-  The Children’s Anxiety Meter-State (CAM-S)  - fear score-  The Child Fear Scale (CFS) |
| **CONCLUSION** | This study conducted in the Pediatric Hematology-Oncology population  reveals the usability of VR distraction. |

| Gold et al. 2021 | |
| --- | --- |
| **STUDY DESIGN** | Prospective RCT |
| **PARTICIPANT** | Patients from aged 8-12 undergoing intravenous (IV) placement. |
| **INCLUSION CRITERIA** | - patients from the hospital's Department of Radiology aged 8-12  - awaiting MRI or CT scans that required IV placement |
| **EXCLUSION CRITERIA** | - cognitive disabilities  - taking pain medication  - negative the cognitive and physical screening results |
| **INTERVENTION ENVIRONMENT** | - immersive VR |
| **INTERVENTION** | 1. Experimental group- standard care + VR (n=10)  - the 5DT HMD 800  - the immersive "Street Luge" game  - the intervention began 5 minutes before the IV placement and ended 5 minutes after the procedure  2. Control group- standard care (n=10)  - an anesthesia spray before the IV placement  - the patients were given the opportunity to play with the VR for 3 min following the completion of their IV placement |
| **OUTCOMES** | 1. Primary outcomes:  - pre-existing pain  - IV pain intensity  - past IV pain intensity  - affective pain intensity  Measured using the Wong-Baker FACES Pain Rating Scale and the Faces Pain Scale–Revised  - anxiety sensitivity- the Childhood Anxiety Sensitivity Index  - sickness feel as a result of the intervention- the Child Simulator Sickness Questionnaire  - child’s engagement with the intervention- the Child Presence Questionnaire  - satisfaction- Likert-format surveys assessing behavioral distress reduction and overall satisfaction for the child, parent and nurse  Only the pain assessment was conducted at three timepoints during the intervention. |
| **CONCLUSION** | This study has demonstrated both the feasibility and utility of VR pain distraction for IV Placement in an outpatient radiology department. |

| Hunder et al. 2022 | |
| --- | --- |
| **STUDY DESIGN** | Crossover - RCT |
| **PARTICIPANT** | Children and adolescents aged 8 to 18 years undergoing  treatment for cancer with upcoming SCP needle insertions. |
| **INCLUSION CRITERIA** | - aged 8 to 18 years;  - able to speak and understand English;  - actively undergoing treatment for cancer;  - at least 1 month, but <3 years, from initial diagnosis;  - requiring at least 2 SCP needle insertions for cancer-related treatment over the following 8 weeks. |
| **EXCLUSION CRITERIA** | - had visual, auditory or cognitive impairments precluding interaction with the intervention (VR) or control (iPad) equipment;  - had major comorbid medical or psychiatric conditions (including needle-phobia) as reported by their health care provider or parent;  - were receiving end-of-life care;  - had a methicillinresistant Staphylococcus aureus infection or symptoms of respiratory or gastrointestinal infection as reported by any member of their health care team which could contaminate the intervention or control equipment;  - had participated in the prior study examining usability of the VR intervention. |
| **INTERVENTION ENVIRONMENT** | - immersive VR |
| **INTERVENTION** | Experimental group (n=20)  - children wore the VR head-mounted display, noisecancelling headphones (to deliver sound) and held a wireless Bluetooth controller (to interact with the VR environment).  Control group (n=20)  - participants watched a video on an iPad while wearing the same headphones as in the experimental group.  - The active control participants watched the same video of an underwater environment with sea creatures and listened to the same music. |
| **OUTCOMES** | - Pain Intensity: 11-point Numeric Rating Scale (NRS - Fear: Child Fear Scale - Distress: 11-point NRS - Immersiveness: 3-point scale - Pain Catastrophizing: Pain Catastrophizing Scale for Children (PCS-C) - Intervention Satisfaction and Acceptability: 4-point scale from “not at all” to “very much.” |
| **CONCLUSION** | - VR as a distraction intervention was feasible and acceptable to patients, their families, and clinicians. |

| Sabel et al. 2016 | |
| --- | --- |
| **STUDY DESIGN** | Crossover RCT |
| **PARTICIPANT** | Children aged 7–17, brain tumor survivors. |
| **INCLUSION CRITERIA** | -age 7–17 years  -completed treatment including RT,  -brain tumour between 1 and 5 years earlier, |
| **EXCLUSION CRITERIA** | -medical condition making them unable to follow the study protocol, e.g. uncontrolled seizures, severe motor or visual impairment or autism,  -were receiving tumour treatment,  -were not speaking Swedish |
| **INTERVENTION ENVIRONMENT** | Console, AVG |
| **INTERVENTION** | 1. Experimental conditions and control conditions for both groups. One group was on the waiting list first and received the intervention later, the other group received the intervention first, and was moved then to the control conditions.   Intervention:   - Nintendo Wii based - 30 min per day activity, 5 times a week - 10-12 weeks - Games with physical activity, balance and - gross body movements required |
| **OUTCOMES** | 1. Primary outcomes:   -physical activity – SenseWear Armband  -physical functioning - Bruininks–Osteretsky Test of Motor  Performance, Second Edition (BOT-2)        2. Secondary outcomes:  -following the instructions – online coaching sessions |
| **CONCLUSION** | In this group of childhood brain tumour survivors, home-based AVG, supported by a coach, was a feasible, enjoyable and moderately intense form of exercise that improved Body Coordination. |

| Sabel et al. 2017 | |
| --- | --- |
| **STUDY DESIGN** | Crossover RCT |
| **PARTICIPANT** | - Children 7 to 17 years old diagnosed with a brain tumor  - Diagnosed after 2003 who had  - completed treatment, including radiotherapy. |
| **INCLUSION CRITERIA** | - children 7 to 17 years old,  - diagnosed with a brain tumor after 2003,  - completed brain tumor treatment (including radiotherapy) 1 to 5 years. |
| **EXCLUSION CRITERIA** | - not in clinical remission or stable disease (ongoing treatment),  - in a medically unstable situation or suffering from another medical condition making them unable to follow the study protocol (eg, severe mental retardation, severe autism, photosensitive seizures),  - not Swedish-speaking. |
| **INTERVENTION ENVIRONMENT** | Non – immersive VR (active video gaming) |
| **INTERVENTION** | - active video gaming,  - using a motion-controlled video console (Nintendo Wii),  - for a minimum of 30 minutes a day, at least 5 days per week, for 10 to 12 weeks  - weekly Internet-based coaching sessions. |
| **OUTCOMES** | - Cognitive Assessment: cognitive tests  - Execution of Activities of Daily Living: he Assessment of Motor and Process Skills (AMPS) |
| **CONCLUSION** | Active video gaming used as a home-based intervention for childhood brain tumor survivors improved motor and process skills in activities of daily living. |

| Semerci et al. 2020 | |
| --- | --- |
| **STUDY DESIGN** | Prospective RCT |
| **PARTICIPANT** | Patients from aged 5-12 undergoing blood draw. |
| **INCLUSION CRITERIA** | - patients aged 5-12  -  undergoing blood draw |
| **EXCLUSION CRITERIA** | -  aged <5 and >12 years  -   chronic or genetic diseases  -   visual problem |
| **INTERVENTION ENVIRONMENT** | - immersive VR |
| **INTERVENTION** | 1. Experimental group- VR (n=35)  -  Samsung Gear Oculus headset  - VR-Rollercoaster  2. Control group- standard care (n=36) |
| **OUTCOMES** | 1. Primary outcomes:  -  patient-reported pain scores  - parent-reported pain scores  Measured using the Wong-Baker FACES (WBS) Pain Rating Scale.  -  patient-reported anxiety score  -  parent-reported anxiety score  Measured using the Children's Anxiety Meter.  -  patient-reported fear score  -  parent-reported fear score  Measured using the Child Fear Scale. |
| **CONCLUSION** | The use of VR for children receiving blood draw is an effective nonpharmacological method to decrease pain, fear and anxiety. |

| Sharifpour et al. 2021 | |
| --- | --- |
| **STUDY DESIGN** | RCT |
| **PARTICIPANT** | Adolescents 14–18 years old |
| **INCLUSION CRITERIA** | - Diagnosed with cancer (osteosarcoma, Ewing's sarcoma, brain tumours, ovarian cancer and skeletal muscle cancer) - undergoing chemotherapy, - 14–18 years old, - informed consent (the patients' parents, the patients and their physicians). |
| **EXCLUSION CRITERIA** | No data |
| **INTERVENTION ENVIRONMENT** | - immersive VR |
| **INTERVENTION** | Experimental group (n=15)   - underwent eight 30-min sessions of VRT (stroll along the beach and a journey to the depths of the ocean) once a week for 2 months.   Control group (n=15)   - did not receive any intervention and were put on a waiting list. |
| **OUTCOMES** | - Pain, Pain-related anxiety or fear: McGill Pain Questionnaire, Pain anxiety symptoms scale, Pain catastrophizing scale, Pain self-efficacy questionnaire (PSEQ). |
| **CONCLUSION** | - Virtual reality can improve pain-related variables among adolescents with cancer during chemotherapy. |

| Wong et al. 2021 | |
| --- | --- |
| **STUDY DESIGN** | RCT |
| **PARTICIPANT** | Pediatric cancer patients aged 6 to 17 years |
| **INCLUSION CRITERIA** | - age between 6 and 17 years, - scheduled to receive PIC, - ability to communicate in Chinese. |
| **EXCLUSION CRITERIA** | - had cognitive and learning difficulties, - had a history of seizures and motion sickness, - were previously diagnosed to have brain tumors or metastasis, - were known to be on contact precaution. |
| **INTERVENTION ENVIRONMENT** | - immersive VR |
| **INTERVENTION** | Intervention group (n=54) VR + standard care  -received virtual reality distraction intervention - 2 animated videos from “Minions” were selected (5 minutes before and during PIC).  -standard care.  Control group (n=54)  -received standard care |
| **OUTCOMES** | Primary Outcome   - Pain: Visual analog scale   Secondary Outcomes   - Anxiety: The state anxiety scale for children - Pulse rate: standard pulse-oxygen monitor - Length of pic procedure: a standard stopwatch |
| **CONCLUSION** | Using VR in clinical setting is feasible and effective in reducing pain and anxiety among pediatric patients undergoing PIC. |
